# Association between variants in inflammation and cancer-associated genes and risk and survival of cholangiocarcinoma

**DOI:** 10.1002/cam4.501

**Published:** 2015-08-15

**Authors:** Roongruedee Chaiteerakij, Brian D Juran, Mohammed M Aboelsoud, William S Harmsen, Catherine D Moser, Nasra H Giama, Loretta K Allotey, Teresa A Mettler, Esha Baichoo, Xiaodan Zhang, Terry M Therneau, Konstantinos N Lazaridis, Lewis R Roberts

**Affiliations:** 1 Division of Gastroenterology and Hepatology, Mayo Clinic College of Medicine, and Mayo Clinic Cancer CenterRochester, Minnesota; 2 Department of Medicine, Faculty of Medicine, Chulalongkorn University and King Chulalongkorn Memorial Hospital, Thai Red Cross SocietyBangkok, Thailand; 3 Department of Biomedical Statistics and Informatics, Mayo Clinic College of Medicine, and Mayo Clinic Cancer CenterRochester, Minnesota

**Keywords:** Bile duct cancer, biliary tract cancer, gene variant, genetic susceptibility, risk factor

## Abstract

Genetic risk factors for cholangiocarcinoma (CCA) remain poorly understood. We assessed the effect of single-nucleotide polymorphisms (SNPs) of genes modulating inflammation or carcinogenesis on CCA risk and survival. We conducted a case-control, candidate gene association study of 370 CCA patients and 740 age-, sex-, and residential area-matched healthy controls. Eighteen functional or putatively functional SNPs in nine genes were genotyped. The log-additive genotype effects of SNPs on CCA risk and survival were determined using logistic regression and the log-rank test, respectively. Initial analysis identified significant associations between SNP rs2143417 and rs689466 in cyclooxygenase 2 (*COX-2*) and CCA risk, after adjusting for multiple comparisons (cutoff of *P *=* *0.0028). However, these findings were not replicated in another independent cohort of 212 CCA cases and 424 matched controls. No significant association was found between any SNP and survival of CCA patients. Although *COX-2* has been shown to contribute to cholangiocarcinogenesis, the *COX-2* SNPs tested were not associated with risk of CCA. This study shows a lack of association between variants of genes related to inflammation and carcinogenesis and CCA risk and survival. Other factors than these genetic variants may play more important roles in CCA risk and survival.

## Introduction

Cholangiocarcinoma (CCA), or bile duct cancer, is the second most common primary liver cancer. The genetic and molecular bases of CCA are not fully understood. In addition to alterations in oncogenes and tumor suppressor genes, chronic inflammation, typified by up-regulation of the inflammation-related genes, cyclooxygenase 2 (*COX-2*) and interleukin-6 (*IL-6*), has been shown to promote CCA growth 1. We hypothesized that genetic variants in genes associated with inflammation and carcinogenesis determine risk and survival of CCA. We examined the association between known or putative functional single-nucleotide polymorphisms (SNPs) of inflammation or cancer-associated genes and CCA risk or survival. We also attempted to validate the reported relationships between natural killer cell receptor group 2 member D (*NKG2D*) gene variants and primary sclerosing cholangitis (PSC)-induced CCA 2.

## Methods

### Patient cohort

We conducted a case-control, candidate gene association study of 370 CCA patients and 740 healthy controls. All patients were seen at Mayo Clinic, Rochester, MN and provided consent and blood DNA samples for IRB-approved research. The diagnosis of CCA was made using previously described criteria 3. Three hundred and seventy CCA patients seen between 2000 and 2010 were included in the discovery study; a second independent cohort of 212 CCA cases seen between 2011 and 2014 were used for validation. Participants in the Mayo Clinic Genome Consortia (Mayo GC) with no previous history of cancer were 2:1 matched to cases by age (±5 years), sex and residential area and used as controls. Details of the Mayo GC cohort were well-described elsewhere 4.

For validation of the published observation of increased CCA risk conferred by *NKG2D* variants, a third control cohort comprising 183 PSC patients without CCA seen between 2004 and 2008 was used 5.

### SNP selection and genotyping

Nine genes, including *COX-2*, *IL6*, interleukin-6 receptor (*IL6R*), interleukin-6 signal transducer (*IL6ST*), glutathione *S*-transferase omega-1 (*GSTO1*), sulfatase 1 (*SULF1*), vascular endothelial growth factor A (*VEGFA*), WD repeat containing, antisense to TP53 (*WRAP53*), and *NKG2D*, were selected based on their known or implied functions in CCA pathogenesis. Eighteen functional or regulatory SNPs in the nine genes were selected (Table1) based on the following criteria: (1) minor allele frequency >5%; (2) previous association with CCA or other malignancies; and (3) known or predicted function using the Function Analysis and Selection Tool for SNPs 6.

**Table 1:** Analysis of association between minor allele frequency of the SNPs studied and cholangiocarcinoma risk in CCA cases versus Mayo GC controls

Genes	SNP ID	Minor allele	Discovery cohort(370 CCA cases vs. 740 Mayo GC controls)			Validation cohort(212 CCA cases vs. 424 Mayo GC controls)		
			Frequency of minor allele between cases vs. controls	OR (95% CI)	*P*^1^	Frequency of minor allele between cases vs. controls	OR (95% CI)	*P*
*COX2*	rs689466	C	0.22 vs. 0.17	1.36 (1.10–1.69)	0.005	0.19 vs. 0.18	1.08 (0.80 to–1.43)	0.64
	rs11583191	A	0.14 vs. 0.12	1.23 (0.95–1.60)	0.12			
	rs2143416	C	0.16 vs. 0.14	1.18 (0.93–1.51)	0.18			
	rs2143417	T	0.20 vs. 0.14	1.52 (1.21–1.91)	0.0003	0.15 vs. 0.15	1.04 (0.75–1.45)	0.79
	rs2745559	A	0.15 vs. 0.17	0.86 (0.67–1.11)	0.25			
*IL6*	rs1800797	A	0.40 vs. 0.43	0.86 (0.72–1.03)	0.097			
	rs2069832	A	0.41 vs. 0.44	0.88 (0.73–1.05)	0.14			
	rs2069837	G	0.07 vs. 0.07	0.93 (0.66–1.32)	0.70			
*IL6R*	rs8192282	A	0.16 vs. 0.16	1.00 (0.79–1.28)	0.99			
*IL6ST*	rs2112979	G	0.28 vs. 0.27	1.05 (0.86–1.28)	0.62			
	rs6870870	A	0.41 vs. 0.40	1.01 (0.85–1.20)	0.91			
*GSTO1*	rs4925	A	0.34 vs. 0.32	1.11 (0.92–1.34)	0.28			
*SULF1*	rs16935901	C	0.13 vs. 0.14	0.97 (0.75–1.26)	0.82			
	rs2725092	G	0.19 vs. 0.20	0.96 (0.77–1.19)	0.69			
*VEGFA*	rs2010963	C	0.30 vs. 0.33	0.88 (0.73–1.07)	0.20			
*WRAP53*	rs2287497	A	0.14 vs. 0.10	1.37 (1.05–1.78)	0.0195			
*NKG2D*	rs11053781	T	0.49 vs. 0.50	0.95 (0.80–1.14)	0.60			
	rs2617167	A	0.23 vs. 0.26	0.83 (0.67–1.02)	0.078			

SNPs, single-nucleotide polymorphisms; CCA, cholangiocarcinoma; Mayo GC, Mayo Clinic Genome Consortia; OR, odds ratio; *COX-2*, cyclooxygenase 2; *IL6*, interleukin-6; IL6R, interleukin-6 receptor; IL6ST, interleukin-6 signal transducer; *GSTO1*, glutathione *S*-transferase omega-1; *SULF1*, sulfatase1; *VEGFA*, vascular endothelial growth factor A; *WRAP53*, WD repeat containing, antisense to TP53; *NKG2D*, natural killer cell receptor group 2 member D.

1 The level of significance was 0.0028 after adjusting for multiple comparison.

All 18 SNPs were genotyped in the 370 discovery cases using Taqman Assays (Applied Biosystems, Foster City, CA). The two significant SNPs from the discovery cohort were validated in the 212 CCA case cohort. The two *NKG2D* SNPs were genotyped in the third cohort of 183 PSC controls. The call rates were between 97.8% and 100%. Two percent of the samples were randomly selected and re-genotyped to assess the genotyping quality with concordance of >99%.

For the Mayo GC controls, genotype data were obtained from the Mayo GC database comprising 14 genome-wide association studies conducted at Mayo 4. Details of the harmonization of control genotypes generated using multiple platforms, standard quality control metrics, and genome-wide imputation were described elsewhere 4.

### Statistical analysis

Hardy–Weinberg equilibrium was examined. The impact of SNP on CCA risk was assessed using univariate unconditional logistic regression under a log-additive model. Association with survival, calculated from the first Mayo visit date to the last follow up or death date, was assessed using Cox Proportional Hazards regression. The Bonferroni correction was used to adjust for multiple comparisons; the cut-off *P* values were 0.0028 for the 18 SNPs in the case—control study and 0.025 for the case—PSC control analyses, respectively.

## Results

Two hundred and sixteen (58%) of the 370 CCA cases and 434 (59%) of the 740 Mayo GC controls were male. The mean ages (±SD) of cases and controls were 60.3 (±13.0) and 60.4 (±13.0), respectively. There were 335 (90.5%) white, 1 (0.3%) nonwhite, and 34 (9.2%) unknown race subjects in the case group; and 716 (96.8%) white and 24 (3.2%) unknown race subjects in the control group (Table2).

**Table 2:** Baseline characteristics of study cohort

Variables	Discovery cohort		Validation cohort	
	370 CCA cases	740 controls	212 CCA cases	424 controls
Age (mean ± SD)	60.3 ± 13.0	60.4 ± 13.0	62.8 ± 13.0	63.9 ± 12.0
Male	216 (58.4%)	434 (58.6%)	126 (59.4%)	249 (58.7%)
Race				
White	335 (90.5%)	716 (96.8%)	212 (100%)	397 (93.6%)
Nonwhite	1 (0.3%)	0 (0%)	0 (0%)	2 (0.5%)
Unknown	34 (9.2%)	24 (3.2%)	0 (0%)	25 (5.9%)
Primary sclerosing cholangitis	72 (19.5%)	12 (1.6%)	50 (23.6%)	3 (0.7%)
Cirrhosis	22 (5.9%)	45 (6.1%)	25 (11.8%)	13 (3.1%)
Diabetes	53 (14.3%)	92 (12.4%)	41 (19.3%)	65 (15.3%)
Smoking	190 (51.4%)	464 (62.7%)	101 (47.6%)	320 (75.5%)
Chronic hepatitis C infection	5 (1.4%)	7 (0.9%)	4 (1.9%)	2 (0.5%)

CCA, cholangiocarcinoma.

Fifty-four (75%) of the 72 CCA with PSC cases and 107 (58%) of the 183 PSC controls were male (*P* = 0.01). There were 71 (99%) white, and 1 (1%) unknown race in the CCA with PSC cases and 178 (97%) white, 4 (2%) nonwhite and 1 (1%) unknown race in the PSC controls. The mean age (±SD) of CCA with PSC cases was 47.7 (±11.2) versus 51.2 (±14.8) years for PSC controls (*P* = 0.04). Because age and sex were significantly different between the two groups, they were included in the statistical model to examine the association between *NKG2D* variants and CCA risk among PSC patients.

The discovery analysis identified a significant association of *COX-2 r*s2143417 and a borderline association of *COX-2* rs689466 with CCA risk (Table1). These two associations were not replicated in the validation cohort. No significant association was identified between the tested *NKG2D* SNPs and CCA risk among PSC patients (data not shown). Similarly, no significant associations were found between the frequencies of either the alleles or the genotypes of any of the tested SNPs and survival of CCA patients (data not shown). When classified by subtypes as intrahepatic and extrahepatic CCA, the results remained consistent.

## Discussion

Our findings do not support a role for selected variants in inflammation and cancer-associated genes in CCA risk and survival, although these genes were selected a priori based on their known or predicted functions in cholangiocarcinogenesis. Despite the use of an a priori selection approach to minimize the possibility of detecting significant SNPs by chance, our findings suggest that the significant SNPs identified in the discovery cohort were likely false-positive observations. The baseline characteristics of the discovery and validation cohorts were comparable. It is unlikely that the findings were not replicated due to inadequate power as the minor allele frequencies of the two validating SNPs in the case and control groups were almost identical. Our findings suggest that other SNPs in the selected genes or in other genes not tested in this study may be more important risk variants. A comprehensive study using a genome-based approach is required to determine significant SNPs that contribute to CCA risk.

A major strength of this study was that it is one of the largest studies so far investigating genetic risk factors for CCA. Second, we performed a validation study of our findings. Third, we performed a replication study of SNPs previously shown to be associated with CCA, particularly investigating associations between the two NKG2D SNPs and cholangiocarcioma that have never been replicated either within the original study or by other groups. Finally, our approach to analysis was conservative, using the Bonferroni correction for multiple testing. The main limitation of this study was that, although CCA is currently classified into three distinct CCA subtypes as intrahepatic, perihilar and distal CCA, because of the limited number of cases of each CCA subtype, we were not able to perform separate analyses for each CCA subtype. Nonetheless, we had sufficient statistical power for an analysis in which we categorized CCA patients into two groups as intrahepatic and extrahepatic CCA. The subgroup analyses of the two CCA subtypes did not reveal any significant associations between SNPs and CCA risk, thus confirming our main findings. In order to separately evaluate genetic risk factors for the three CCA subtypes, an expansion of the CCA patient cohort is required. A collaborative effort among several investigators for germline DNA sample accrual is currently underway.

## Conflict of Interest

The contents are solely the responsibility of the authors and do not necessarily represent the official views of the National Institutes of Health.
